# Deep Learning for Opportunistic Rain Estimation via Satellite Microwave Links

**DOI:** 10.3390/s24216944

**Published:** 2024-10-29

**Authors:** Giovanni Scognamiglio, Andrea Rucci, Attilio Vaccaro, Elisa Adirosi, Fabiola Sapienza, Filippo Giannetti, Giacomo Bacci, Sabina Angeloni, Luca Baldini, Giacomo Roversi, Alberto Ortolani, Andrea Antonini, Samantha Melani

**Affiliations:** 1 MBI Srl, 56121 Pisa, Italy; arucci@mbigroup.it (A.R.); avaccaro@mbigroup.it (A.V.); 2 National Research Council of Italy, Institute of Atmospheric Sciences and Climate (CNR-ISAC), 00133 Rome, Italy; elisa.adirosi@artov.isac.cnr.it (E.A.); sabina.angeloni@artov.isac.cnr.it (S.A.); l.baldini@isac.cnr.it (L.B.); g.roversi@isac.cnr.it (G.R.); 3 Dipartimento Ingegneria dell’Informazione, University of Pisa, 56122 Pisa, Italy; fabiola.sapienza@ing.unipi.it (F.S.); filippo.giannetti@unipi.it (F.G.); giacomo.bacci@unipi.it (G.B.); 4 Laboratory of Environmental Monitoring and Modelling for the Sustainable Development (LaMMA), 50019 Florence, Italy; ortolani@lamma.toscana.it (A.O.); antonini@lamma.toscana.it (A.A.); melani@lamma.toscana.it (S.M.); 5 National Research Council of Italy, Institute for BioEconomy (CNR-IBE), 50019 Florence, Italy

**Keywords:** disdrometer, rain sensors, opportunistic sensors, sensor networking, artificial intelligence, multi-sensor big data

## Abstract

Accurate precipitation measurement is critical for managing flood and drought risks. Traditional meteorological tools, such as rain gauges and remote sensors, have limitations in resolution, coverage, and cost-effectiveness. Recently, the opportunistic use of microwave communication signals has been explored to improve precipitation estimation. While there is growing interest in using satellite-to-earth microwave links (SMLs) for machine learning-based precipitation estimation, direct rainfall estimation from raw signal-to-noise ratio (SNR) data via deep learning remains underexplored. This study investigates a range of machine learning (ML) approaches, including deep learning (DL) models and traditional methods like gradient boosting machine (GBM), for estimating rainfall rates from SNR data collected by interactive satellite receivers. We develop real-time models for rainfall detection and estimation using downlink SNR signals from satellites to user terminals. By leveraging a year-long dataset from multiple locations—including SNR measurements paired with disdrometer and rain-gauge data—we explore and evaluate various ML models. Our final models include ensemble approaches for both rainfall detection and cumulative rainfall estimation. The proposed models provide a reliable solution for estimating precipitation using Earth–satellite microwave links, potentially improving precipitation monitoring. Compared to the state-of-the-art power-law-based models applied to similar datasets reported in the literature, our ML models achieve a 46% reduction in the root mean squared error (RMSE) for event-based cumulative precipitation predictions.

## 1. Introduction

Real-time and precise precipitation measurement is of great interest for decision makers in a wide range of applications. It plays a pivotal role in influencing agricultural outcomes, and precipitation is a key farming weather indicator, enhancing crop quality and economizing on energy and water, paving the way for Agriculture 4.0 [1]. Rainfall data also serve as a significant reference for reservoir management, city water provisions, irrigation strategies, and broader water resources planning [2], as well as for energy strategizing [3]. However, capturing precise rainfall data in real-time remains arduous due to their extensive spatial and temporal variations [4]. Moreover, the detailed tracking of ground-level precipitation across expansive terrains is imperative for climatic and global warming studies [5]. Numerous terrestrial regions suffer from a quasi-total lack of accurate rainfall measurements, particularly ground measurements at daily or sub-daily intervals [6].

For these reasons, over the last several years, there has been a growing demand for more advanced and refined methods for monitoring precipitation on a broad geographical scale. This increased focus stems from a need to meet several critical criteria: higher accuracy in quantifying precipitation rates; a sufficient spatial distribution of sensors across a given area; uninterrupted spatio-temporal data availability; and the ability to promptly generate detailed precipitation maps for near-real-time applications [7,8].

The traditional means of precipitation measurement have been unable to fully meet these criteria. These conventional systems typically involve a network of rain gauges, weather radars, and satellite sensors. Despite their capabilities, none of these traditional systems fully satisfy the aforementioned stringent requirements [6]. Hence, in the past decade, research into non-traditional rain rate (RR) estimation methods has gained traction, and the use of alternative near-surface rainfall data sources has been extensively considered. Indeed, a wide range of sensors can be used opportunistically for rainfall estimation: windshield wipers [9], crowd-sourced personal weather stations using the internet of things (IoT) [10], and terrestrial and satellite broadcast receivers.

These novel techniques focus on quantifying rain-induced attenuation in existing radio communication networks, which include both cellular backhaul connections and satellite-to-earth microwave links (SMLs), particularly those operating in Ku and Ka frequency bands [11,12,13,14,15]. While commercial microwave links (CMLs) for rain estimation are well-studied [16,17,18,19,20,21,22,23,24], the field of Earth-to-satellite links is still nascent, and further research is warranted given the limitations due to their scarce coverage [4]. Standard techniques, often based on physical models and heuristic methods, can estimate local RR at the receiver site if certain meteorological and geometric parameters are known. Such techniques can also partner with traditional tools, e.g., pairing SMLs to back regional rain gauges (RGs) and weather radar monitoring systems [11,25].

However, these traditional approaches often lack reliability and scalability and fall short on meeting the demanding criteria for accuracy, spatial coverage, data availability and real-time applicability [6]. Recent studies have begun to explore machine learning (ML) for this application, e.g., deep learning (DL) techniques for rain estimation with CMLs using recurrent neural network (RNN) [26,27,28], but, in the case of satellite data, they remain preliminary and address only the detection task of identifying rain samples [29,30,31,32]. To date, no published research has developed an end-to-end ML model for estimating RR from raw SML data.

In this regard, the main problem this research aims to address is the potential of ML methods for real-time, reliable, and scalable precipitation measurement using SMLs. Given the preliminary successes of ML techniques in related domains, there is a pressing need to also investigate the capability and efficacy of these models for rainfall estimation in this case. In particular, the objectives of this work are to develop ML models for both rainfall detection—to effectively classify whether rain is occurring based on raw SML data—and the estimation of the amount of precipitation, focusing in particular on real-time inference.

The paper is structured as follows. In Section 2, a literature review is conducted, focusing on rainfall estimation, particularly emphasizing the current research and state-of-the-art in satellite link rainfall inversion. Next, we detail our experimental setup in Section 3, describing the data collection process, the details of the ML methodology employed, and the data preprocessing steps. Then, in Section 4, we show our results, systematically exploring the procedures used to identify the optimal models for our objectives, eventually assessing our best-performing model on a test set with a focus on cumulative rainfall prediction.

## 2. Background

The opportunistic use of sensors not primarily built to detect rain aligns with the growing demand for environmental opportunistic sensing and has been extensively researched in recent years. The core concept of these methods is to evaluate the total attenuation caused by rainfall on a received signal [33], e.g., terrestrial links like cellular network backhaul connections, or satellite-to-earth. Knowledge about the signal attenuation can directly be linked to rainfall by a power-law relationship, as deeply explored in numerous works [13,15,34,35,36]. Later works have also shown how to enhance the scalability of these systems, making them ideal for real-world applications by addressing the limitations inherent in CMLs or non-interactive SML systems [12,29].

### 2.1. Traditional SML-Based Rainfall Inversion Method

Since the ML methods proposed in this paper are strictly related to the mechanism and the methodologies applied in the traditional opportunistic rainfall estimations, we summarize here the key points, referring to the work in [11,37].

**Figure 1:**
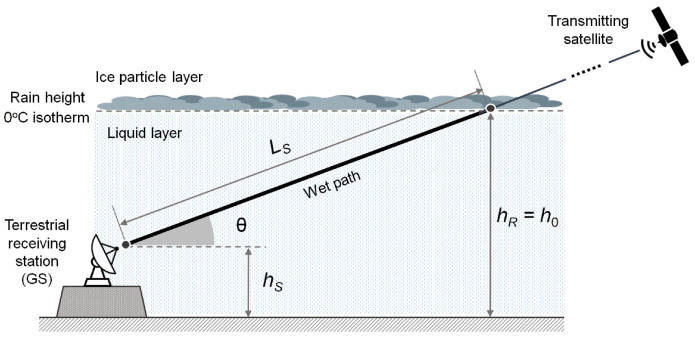
Satellite downlink scheme in the case of precipitation. Picture taken from [11].

The downlink from a satellite to a user terminal (UT) under stratiform rain conditions is illustrated in Figure 1. The satellite signal passes through two layers: the top *ice particles layer (IPL)*, primarily consisting of ice as dry frozen particles, and the bottom *liquid layer (LL)*, marked by liquid-phase precipitation, separated by the rain origin height at $h_{R}$, equal to the 0 °C isotherm at $h_{0}$. While the former has negligible contribution to the attenuation, the latter has a pronounced effect on the received signal UT at altitude $h_{s}$ and elevation angle θ from the horizon. Signal quality is conveyed by the signal-to-noise ratio (SNR), which is the power ratio *C* of the useful signal to the noise power *N* over bandwidth *B*, both assessed at the receiving antenna. In particular, the signal quality is given by the average energy per symbol $E_{s}$ received at radio frequency (RF) relative to the noise power spectral density $N_{0}$, known as energy-per-symbol-to-noise density ratio (ESNDR) and noted as $E_{s}$/$N_{0}$.

Under clear skies, the median received ESNDR $E_{s}/N_{0}$ is roughly 10.5dB [34]. When it rains, the ESNDR signal attenuation is depicted by the specific logarithmic attenuation *k* (in dB/km). It is empirically connected to the RR *R* by the power-law [16]

(1) $$\begin{array}{c} k=aR^{b}\;(1\;\mathrm{dB}/\mathrm{km}) \end{array}$$

where *a* and *b* are coefficients that largely depend on various parameters such as carrier frequency, temperature, or rain angle to the signal path (please see [34] and references therein for additional details). Rainfall rate estimation methods rely on monitoring the received signal performance measure (signal power, SNR, or ESNDR) and gauging its reduction due to rain compared to clear-sky conditions. There are two primary methods for assessing the satellite signal’s attenuation: the first one utilizes the satellite beacon signal, while the second one is based on directly analyzing the signal broadcast by the satellite transponder, where the rainfall rate estimate is based on direct measurements by the receiver. The latter is the one adopted in the work presented in [12], the results of which serve as a benchmark for our ML solution development. In this work, the satellite gateway provides an ESNDR estimate obtained from a bi-directional receiver that is adept at consistently producing $E_{s}/N_{0}$ estimates. The attenuation $L_{\mathrm{rain}}$ due to rainfall is determined as a linear function of the SNR in clear-sky conditions over the SNR in rain conditions:

(2) $$\begin{array}{c} L_{\mathrm{rain}}=\frac{{\left(E_{s}/N_{0}\right)}_{\mathrm{clear}}}{{\left(E_{s}/N_{0}\right)}_{\mathrm{rain}}}\left(1-\epsilon \right)+\epsilon \end{array}$$

where ϵ is a parameter inversely proportional to the attenuation due to other factors $L_{\mathrm{atm}}$ that depends on various atmospheric parameters such as the noise temperature of the atmosphere or the temperature conditions at ground level.

The attenuation $L_{\mathrm{rain}}$ caused by rain on the received signal depends on the specific rain distribution over the wet path of the satellite–ground station (GS) radio path, as shown in Figure 1. Under the assumption that the RR remains fairly steady over this path, the specific attenuation *k* (in dB/km) can be obtained in a straightforward way by dividing the integrated value $L_{\mathrm{rain}}$ by the wet path length. Therefore, with the known values of coefficients *a* and *b*, one can invert (1) and obtain the estimated rainfall rate.

### 2.2. ML Models and Current ML Applications

The ML models used in our analysis are described hereafter:

1. The gradient boosting machine (GBM) builds decision trees sequentially, with each new tree correcting the errors of its predecessors. Each decision tree T consists of nodes N and edges E. Internal nodes represent tests on features $x_{i}$ from the input vector $x=(x_{1},x_{2},\ldots ,x_{n})$, and edges represent the outcomes leading to child nodes. Leaf nodes L⊂N represent the final output value *y*. At each internal node, a feature $x_{i}$ and threshold θ are chosen to split the data: if $x_{i}<\theta$, the left child is followed; otherwise, the right child. GBM constructs an ensemble of shallow trees by fitting the residual errors of prior trees rather than the target variable itself. For a mathematical breakdown of GBM, please refer to [38];
2. A dense neural network (DNN) consists of layers of interconnected neurons with adjustable weights. An input vector $x\in R^{n}$ propagates through the network via weighted sums and nonlinear activation functions. For each layer *l*, the output $h^{\left(l\right)}$ is computed as
  (3) $$\begin{array}{c} h^{\left(l\right)}=\sigma \left(W^{\left(l\right)}h^{\left(l-1\right)}+b^{\left(l\right)}\right), \end{array}$$
  where $W^{\left(l\right)}$ is the weight matrix, $b^{\left(l\right)}$ is the bias vector, and σ is the activation function. The final output y is produced by
  (4) $$\begin{array}{c} y=W^{\left(L\right)}h^{\left(L-1\right)}+b^{\left(L\right)}, \end{array}$$
  where *L* is the output layer index. The network minimizes the error between its predictions and true values using optimization algorithms like gradient descent [39];
3. An RNN processes sequential data by maintaining a hidden state ht that captures information from previous inputs. At each time step *t*, the hidden state is updated based on the current input $x_{t}$ and the previous hidden state ht−1:
  (5) $$\begin{array}{c} ht=f(Wxhxt+Whhht-1+bh), \end{array}$$
  where Wxh is the weight matrix that captures how the current input influences the current hidden state, $W_{hh}$ is the weight matrix that captures the temporal dependencies by incorporating information from previous time steps, bh is a bias term, and *f* is the activation function. The output at time *t* is
  (6) $$\begin{array}{c} y_{t}=g\left(Whyh_{t}+by\right), \end{array}$$
  where Why is the output weight matrix, $b_{y}$ is the bias term, and *g* is the output activation function. Standard RNNs struggle with long sequences due to vanishing or exploding gradients during training [39]. To address this, we use gated recurrent units (GRUs), which employ gating mechanisms to better capture long-range dependencies [40];
4. A multihead neural network (MH-NN) refers to any neural architecture that processes the input vectors through multiple ’heads’—parallel sub-networks—that learn different representations of the data independently before combining them. This design allows the network to learn different representation based on different aspect of the input data. Mathematically, let us decompose the input vector $x\in R^{n}$ in *k* parts. The network processes different x through each head *k* to produce its own hidden representation $h_{k}$:
  (7) $$\begin{array}{c} h_{k}=f_{{\mathrm{head}}_{k}}\left(x;\theta_{k}\right),\;\mathrm{for}\;k=1,2,\ldots ,K, \end{array}$$
  where $f_{{\mathrm{head}}_{k}}$ is the function corresponding to head *k* with its own parameters $\theta_{k}$ and *K* is the total number of heads. Each head can be designed for a specific task or to focus on different characteristics of the data. Eventually, the hidden representations are concatenated and fed as input to a standard DNN to obtain the desired output.

Empirical methods like the one described in Section 2.1 face two main challenges: distinguishing between rainy and non-rainy periods while ignoring other signal variations, and defining the signal baseline, i.e., the clear-sky power level. Accurately determining this baseline is crucial for calculating rain-induced attenuation. While this issue is often addressed by interpolating signals before and after rainfall or employing Kalman tracking systems [12,34], some studies have employed ML to tackle the problem.

In 2013, the authors of [13] were among the first to use a DNN for rainfall detection from a SML, showing promising results. The authors in [14] used logistic regression on extracted bit error rates. In [41], a support vector machine (SVM) model was used to identify rain instances, followed by a recurrent neural network to determine the attenuation baseline. Ref. [42] makes use of a randomized tree classifier for rainfall detection. In [29], a DNN was applied to statistics extracted from the raw attenuation signal to distinguish between dry and wet periods, leveraging the well-established power-law relationship for rainfall estimation. A DNN followed by an empirical model for rainfall inversion has also been proposed in [30].

In [31], the authors used a variety of shallow ML models applied to extracted signal statistics for rainfall detection. They further enhanced detection capabilities with an ensemble model composed of two shallow DNNs in [43]. Alternatively, a long short-term memory (LSTM) architecture for rain event identification has been proposed in [32]. All these studies mainly used ML for classifying dry versus rainy periods and relied on the traditional power-law for rainfall estimation regression, typically using shallow networks with a single hidden layer. To the best of our knowledge, no study has developed an end-to-end DL regression model for rainfall estimation using SMLs.

## 3. Experimental Setup

### 3.1. Data Collection

The data used in this work are sourced from the NEFOCAST platform [44], a project born with the scope of determining the viability of a satellite-based real-time precipitation monitoring system for the Tuscany region in Italy [12,45]. It leverages a comprehensive network of next-gen satellite receivers, named SmartLNB (SLNB). These are interactive satellite terminals that were primarily designed for satellite services, which are used as weather sensors for measuring the rain-induced attenuation of the downlink signal. The measured signal attenuation is then transmitted back in real time via the return channel. The system consists of a multitude of geolocated SLNB satellite receivers, transmitting their measurements to the NEFOCAST Service Center, which functions as a data warehouse. In addition to the SLNB data, the NEFOCAST Service Center also integrates actual rain measurements from external providers, tipping bucket rain gauges (TBRGs), and disdrometers.

The capability to complement observations from the SLNB with actual rainfall measurements distributed across various locations and sources is of great importance for the work presented in this paper, since we can leverage the co-location of the signal receiver and the actual rainfall measurement to train ML models using measured rainfall as the target dependent variable. In particular, the locations with both an SLNB and a co-located rain gauge or disdrometer included the cities of Rome, Pisa, and Massa in Italy (see Table 1).

Given the supervised machine learning nature of the task, we used the satellite SNR time series as input features for the models, while the associated rainfall measurements from the same location served as the target variable. Initially, we attempted to combine data from different precipitation measurement devices to create a more diverse training set. However, the disdrometer and the TBRG could not be combined due to several factors, most notably the different sampling rates—1 min for the disdrometer and 15 min for the TBRG. The TBRG’s lower temporal resolution is due to its underlying mechanism, which requires waiting for the bucket to fill before recording the water amount. This introduces a temporal bias, as the bucket may tip later than when the initial precipitation occurred. Moreover, although one could artificially increase the TBRG’s sampling rate through interpolation, the results would not match the Precision of the disdrometer’s high-resolution measurements. Consequently, we selected the disdrometer as the sole ground-truth target variable, given its much higher sampling frequency compared to the rain gauges. Furthermore, its minute-to-minute precise measurements allow us to train a minute-to-minute detection model. Because the disdrometer was only available in Rome, we used time-series data from the other locations (Pisa and Massa) to analyze the generalization capability of our models. The TBRGs from these other locations were used to assess the model capabilities on event-based cumulative precipitation prediction. Given that the TBRGs values would be summed over the events’ duration, this would effectively remove their temporal bias in the measurement.

### 3.2. Preliminary Analysis

**Figure 2:**
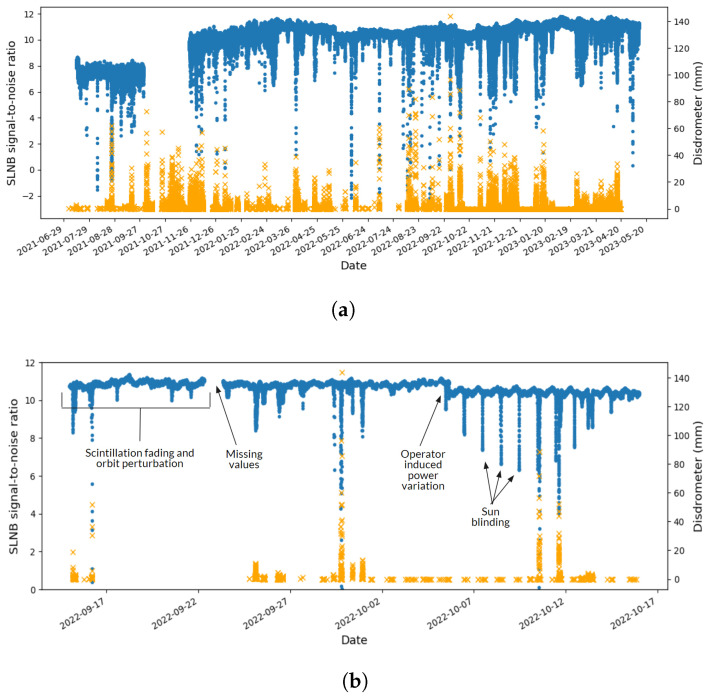
(**a**) Time series of the SNR ratio from the SLNB in the Rome location (blue dots, left axis) and the associated disdrometer rain accumulation measurements (orange crosses, right axis). (**b**) Same view, detailed on a single-month time range and with indications of observed satellite downlink impairments.

We extracted the SLNB time series for the three locations of Rome, Pisa, and Massa, originally sampled at 30 s intervals and interpolated to 1 min intervals. An example of this data in the case of Rome is given in the upper panel of Figure 2. Qualitatively, the SNRs and rainfall time series seem closely synchronized and a prominent linear relation is observed. However, a deeper examination shows that multiple noise sources that hinder the ML task are present (see the bottom panel of Figure 2). These contributions are mostly due to satellite downlink impairments and have been deeply investigated in prior research papers. In particular, these are related to atmosphere interferences, antenna mispointing and orbit perturbations, sun blinding, operator-induced power variations, and extra attenuation due to the rain (we refer to [11] for a detailed description). We therefore implemented a set of preprocessing steps, intended to make the time series ready for model training.

### 3.3. SNR Data Preparation

In order to determine the underlying relationships between the SNR and the actual rainfall measures, we handled missing values—mostly due to outages and server reboot operations—and then applied a *detrending* procedure to clean up the time series from noticeable patterns such as daily cycles and trends. These steps help to improve the ML model capacity to learn the relationship between the predictor and its target, mitigate overfitting, and enhance model stability [46]. The procedure was divided into four steps:

1. *Missing value imputation*. Most intervals with missing values were short, and we used linear interpolation with neighboring values to impute them. We applied this imputation only during non-rain instances to avoid introducing significant bias during concurrent rain events. Linear interpolation was chosen because the SNR remains constant in the absence of rain events, making it suitable for estimating missing values during these periods. Furthermore, interpolation was applied only for brief interruptions in the SNR data (maximum of 5 min); longer periods were left empty and not considered in the training data to prevent potential distortions. Given the short duration of the missing intervals and the stable nature of the SNR during non-rain periods, linear interpolation provided a simple and effective solution with minimal bias introduced;
2. *Outlier removal*. We computed a long-range moving median *q* and discarded values differing by more than xq, i.e., a procedure essentially equivalent to cutting off data exceeding upper and lower quantiles. Boundaries have been chosen through an analysis of the time series along all the time span, leading to the choice of a 120 h window and a value of x=0.06;
3. *Flattening and centering of the SLNB signal*. Short-term linear trend variations, arising from satellite operations where the signal power changes abruptly, causing steps in the data, have been subtracted using a 48 h rolling average of the time series;
4. *Daily cycle removal*. A polynomial function has been used to determine the daily trend, which has been subtracted from the original signal. We employed the mean absolute error (MAE) on the predictions to find the polynomial degree and the time-window that provided the most robust results.

**Figure 3:**
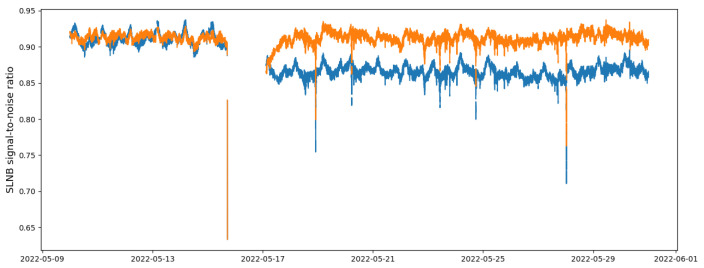
Superposition of original SLNB signal from the Rome location (blue) and post-processed SLNB after the preprocessing steps (orange).

An example of the results obtained after the steps is reported in Figure 3. The processed time series is notably more centered around zero, while preserving the relevant signal variations that are vital for the ML task. It can also be noticed that the sudden steps induced by signal power variations have been removed. Moreover, the analysis of the auto-correlation plots revealed that the 24 h correlation is not significant, i.e., that the daily cycle has been removed.

### 3.4. Disdrometer Data Preprocessing and Rain-Event Selection

Globally, the disdrometer time series was spotless; no missing values nor data corruption were noticed. The main preprocessing required was the definition of a lower threshold. Indeed, the data distribution was incredibly left-skewed with a long tail of insignificantly small values—very minor precipitation or dust that the laser disdrometer interpreted as micro-rainfall droplets. Following the preprocessing steps in [45], which addressed the same disdrometer data, we established a lower threshold of $5\times 10^{-4}\;\mathrm{mm}$ on the target distribution values. This was carried out to exclude the minimal values reported by the disdrometer, as they are likely imperceptible in the SLNB attenuation. While setting a higher threshold could have simplified the classification task, this would have introduced bias into the training data by misclassifying rain events as dry instances. We selected this specific threshold to balance the need to filter out insignificant values with the ability to detect even the slightest drizzle. For context, at a constant rate of $5\times 10^{-4}\;\mathrm{mm}$ per minute, a rain event would need to last approximately 33.3 h to accumulate 1mm of total precipitation.

The identification of the relevant rain events was performed by analyzing the descriptive statistics obtained from the time series. Following the same procedure as in [45], a distinct event of rain is identified as a separation of 30 min between consecutive non-rain instances. Disdrometer data from 2021 to 2023 recorded 653 rain events, despite the great majority of them having actually related to very low rain accumulation. Indeed, the computed median rainfall accumulation of 0.03mm lies below the range of definition of a drizzle [47], i.e., between 0.1mm and 1mm. Therefore, we filtered out cumulative rainfall below this threshold, obtaining 273 events with median 0.88mm and average 2.47mm, with a median event duration of 57 min and an average of 111 min.

**Figure 4:**
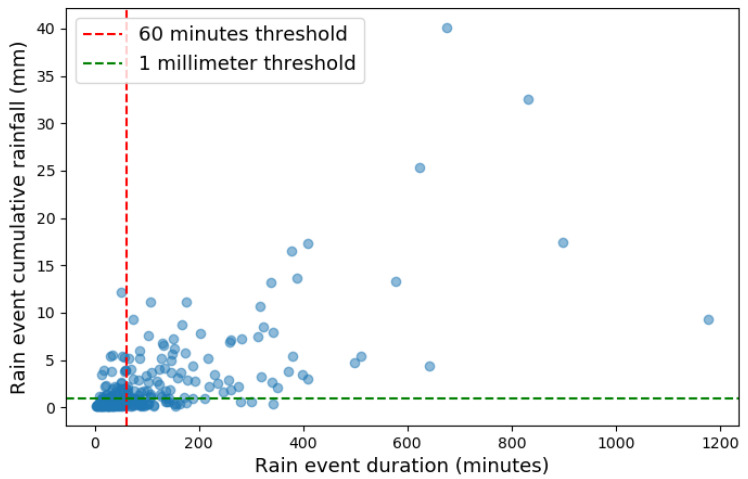
Scatter plot of rain events with threshold at 1mm and 60 min minimum duration.

Our selection criteria resemble the ones used in [45], where a minimum cumulative rainfall of 1mm and a minimum duration of 60 min were used as thresholds. Figure 4 shows the selection process using these criteria, showing an evident linear relationship between cumulative rainfall and event duration. In our data, numerous events with duration under 60 min are associated with cumulative rainfall exceeding 1mm. Conversely, events with cumulative rainfall below 1mm reveal a much more fuzzy relationship between the two variables.

### 3.5. Feature Engineering

We extracted several statistical features from the input data and added them to the model features, resembling the procedure used in similar recent works [30,31]. These include rolling window calculations for the median, standard deviation, and skewness. Furthermore, we defined several functions to determine the local trend, the ratio of singular values, and the frequency energy ratio within the specified time windows of the time series. Statistics have not been restricted to a single time window size, e.g., 30 min as in [31], but considering the values of 2, 5, 20, 60, 360, and 1440 min.

### 3.6. Model Selection

Given the limited literature on ML models for rainfall estimation from satellite downlink attenuation, we primarily embarked on an initial wide screening phase, remaining (as much as possible) assumption-free and exploring a broad range of models generally applied to time-series extrinsic regression and classification tasks. In increasing the order of complexity [39], the set of models we explored included linear models (linear and logistic regressions), tree-based models (random forest [48], k-nearest neighbors (KNN) [49], AdaBoost [50], GBM [38]), and neural networks: DNNs [51], GRU, temporal convolutional networks (TCNs) [52]).

Model selection was performed by adopting a temporal hold-out validation. The full time series from the Rome location, covering the period from mid-2021 to 2023, have been segmented into three parts based on the date: instances before 2023-02-01 were designated as training data, then validation instances were selected until 2023-11-04, and the remaining most recent data were the test set.

Standard area under ROC curve (AUC)–receiver operating characteristic (ROC), F1-score, Precision P, and Recall R [53,54] metrics have been adopted for the classification tasks. Such indicators all range between 0 and 1; an ideal estimator achieves a score equal to 1 for each one of those quantities, which are, respectively, defined as

(8) $$\begin{array}{cc} P= & \frac{T_{p}}{T_{p}+F_{p}} \end{array}$$

(9) $$\begin{array}{cc} R= & \frac{T_{p}}{T_{p}+F_{n}} \end{array}$$

(10) $$\begin{array}{cc} F_{1}= & 2\frac{\mathrm{PR}}{P+R} \end{array}$$

where $T_{p}$, $F_{p}$, and $F_{n}$ are the number of true positives, false positives, and false negatives, respectively, computed by comparing the predicted output $\hat{y}$ provided by the model with the ground-truth labels **y**.

Regarding the regression task, normalized mean absolute error (NMAE) and root mean squared error (RMSE) have been chosen to evaluate the performance of the models:

(11) $$\begin{array}{cc} \mathrm{NMAE}= & \frac{\sum_{i=1}^{n}\left|y_{i}-\hat{y}i\right|}{n(y_{\max}-y_{\min})} \end{array}$$

(12) $$\begin{array}{cc} \mathrm{RMSE}= & \sqrt{\frac{\sum_{i=1}^{n}{\left(y_{i}-{\hat{y}}_{i}\right)}^{2}}{n}} \end{array}$$

where $y_{i}$ represents the actual value, ${\hat{y}}_{i}$ represents the predicted value, *n* is the number of observations, and $y_{\max}$ and $y_{\min}$ are the maximum and minimum values of the actual observations, respectively.

In addition, in order to have a more direct and readable assessment of the results, we also adopted an additional assessment method and compared the cumulative predictions from the regression model with the actual cumulative rainfall for any given rain event in order to assess the error rate on the cumulative precipitation amount on a given rain event. For the latter, assessment metrics include linear correlation coefficient and normalized bias (NB).

Finally, hyperparameter exploration has been performed on each ML model and neural architecture. In particular, a complete grid-search has been applied on non-neural models, while, for neural networks, the tuning of the parameters has been automated using a Bayesian optimization tuning with a Gaussian process [46].

## 4. Results

### 4.1. Detection

In order to explore the capabilities of ML models on the rain-event classification task, a series of preliminary experiments were conducted to test a variety of models and architectures. In general, the performance obtained on the full original dataset, which had more than a 90% imbalance in favor of no-rain instances, was not optimal since trained models tend to favor the dominating class with poor performance on the minority instances. In order to overcome this issue, a random sub-sampling was applied to balance the dataset [55], showing marked improvements. Nevertheless, the best results were obtained by conditioning the dataset to only rain events using the procedure presented in Section 3.

#### 4.1.1. Rain-Conditioned Validation Performance

The best-performing models were a GBM trained solely on the aggregated statistical features, a GRU using the raw past 60 min time-window SLNB input, and an MH-NN trained on both sets of features. The parameters used in the grid search for the GRU and the MH-NN are reported in Table 2 and Table 3, respectively. The final GRU model obtained from the grid search consists of 6 GRU layers with 96 units each and a final DNN layer with 16 units. The average dropout rate per layer is 33%. The final MH-NN, on the other hand, consists of 8 GRU layers and 6 DNN layers forming each “head” and a final DNN layer to combine the output. The details of each layer can be found in Table 3.

We further built an ensemble model [39,46] of the last two classifiers. We initially created an ensemble with all the best-performing models (GBM, GRU, and MH-NN), but we then chose to only include the GRU and MH-NN, as removing the GBM model did not significantly affect the performance. The ensemble was created with a weighted average of the output probabilities of each model. This model performed slightly better with respect to a simple average and no further gain was obtained by previously calibrating the classifiers, as suggested in [46]. In all cases, we used a training–validation dataset imbalance of 50% and a decision threshold optimized for highest F1-score.

**Figure 5:**
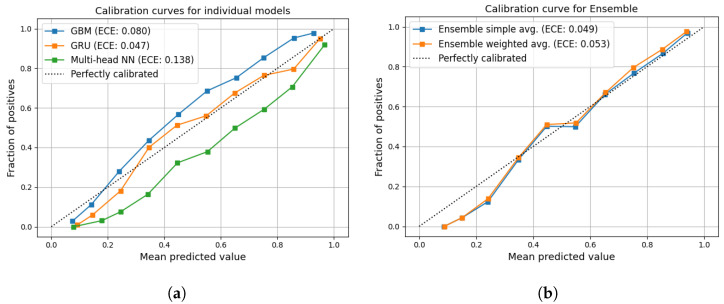
Calibration plots of the best performing models. (**a**) GBM, GRU and MH-NN models. (**b**) ensemble model, with both simple and weighted averages.

The results obtained on the rain-event-conditioned dataset are reported in Table 4. Both the GRU and MH-NN attain comparable performance levels, while the ensemble weighted average achieves better results, with 91.9% AUC–ROC and 81.9% F1-score. Calibration plots are shown in Figure 5, where it can be noticed that the ensemble model exhibits a strong calibration at 0.049. This shows that the ensemble model manages to leverage the inference capabilities of the two individual models whilst keeping the calibration error close to the best individual model error (GRU, 0.047). Comparing the ensemble calibration curve with the single models’ curves, it is evident that the misalignment transitioned from the center towards the lower probability range, suggesting that we effectively reduced the model uncertainty for mid-tier true probability instances, but this improvement was counterbalanced by increased misalignment in the lower true probability spectrum, between 0 and 30%.

#### 4.1.2. Imbalanced Validation Performance

In view of the results obtained on the rain-conditioned dataset, we tested the performance of our pre-trained best models (obtained in Section 4.1.1) on the original validation dataset which has a 91% imbalance in favor of no-rain instances. The results are shown in Table 5. The GBM obtained an AUC-ROC of 0.946 whilst the GRU and MH-NN reached 0.968. These AUC-ROC values are a slight improvement compared to ∼91% obtained in Table 4 with the balanced dataset. In contrast, F1-score values lowered from an average of ∼80% to ∼71%. This effect was expected since a higher class imbalance inevitably leads to a lower Precision score which, in turn, hampers the F1-score.

#### 4.1.3. Decision Threshold Selection

The decision threshold used in the classification task plays a relevant role in the trade-off between the Precision and the Recall performance. In order to quantify the extent to which its value can affect the results, we focused on the best-performing model, the ensemble classifier, and studied the performance obtained under different thresholds by fixing the Recall at various desired levels. Results are reported in Table 6, where three levels have been considered, each of them corresponding to a certain value of the decision threshold. The observed interplay of the Recall–Precision trade-offs suggests that, in a real-world application and depending on the specific requirements, one could effectively vary the threshold in order to maximize the performance towards one of the two metrics, favoring, respectively, smaller false positive rate (FPR) values or higher Precision. In general, both Recall and FPR are unaffected by variations in the class imbalance [54]; therefore, given the dynamic nature of rainfall, where class-balance fluctuates across the year, a fixed threshold would guarantee consistent Recall performance on these metrics, letting Precision vary based on the actual probability of rainfall. We settled on a decision threshold of 65%, as it provided the highest overall F1-score on the validation data (71%) between the three Recall levels considered in Table 6.

#### 4.1.4. Model Assessment on Test Data

**Figure 6:**
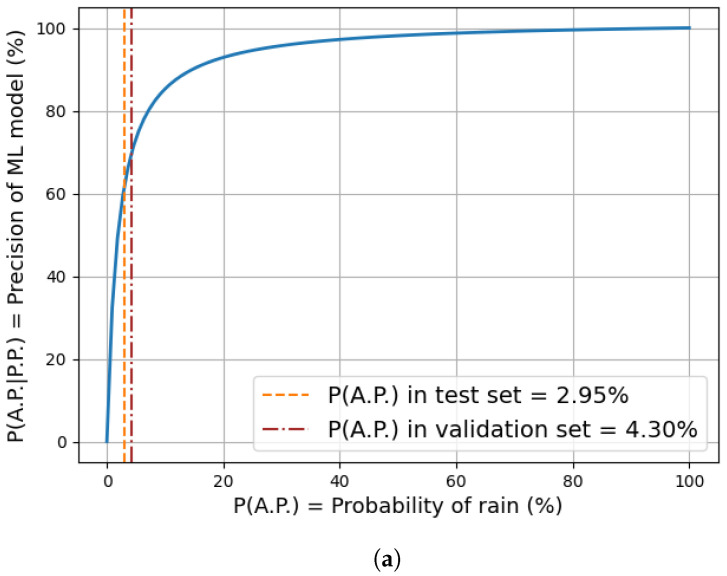

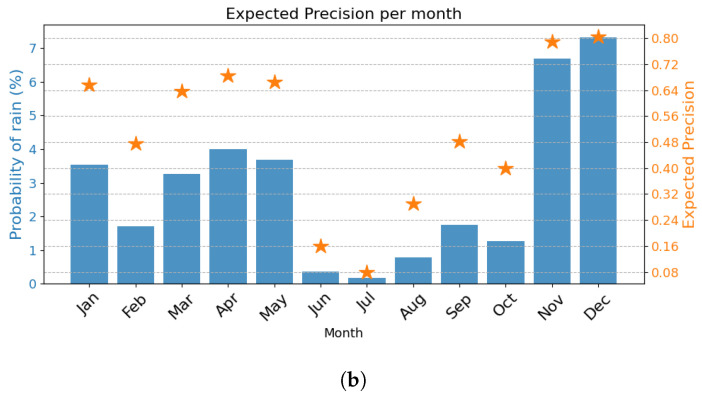
(**a**) Model Precision as a function of positive instance probability (blue line) and actual values obtained from the test (orange dashed segment) and validation datasets (red dashed segment). (**b**) Monthly-based aggregation of model expected Precision.

The performance obtained using our best performing model, the ensemble classifier on the test set, is reported in Table 7. This test dataset has a higher imbalance (97.05%) with respect to that of the validation set, due to the fact that this time frame included mostly the summer months with less precipitation. The results are in line with those obtained on the validation dataset. Finally, in Figure 6, we show the behavior of the Precision performance with respect to the actual probability of observing a rain instance. Precision is a class-balance sensitive metric since it is computed by conditioning on predicted positives. This conditioning negatively affects Precision in cases of high imbalance. Indeed, applying the Bayes theorem to the Precision metric, we obtain

(13) $$\begin{array}{cc} P(\mathrm{AP}|\mathrm{PP})= & \frac{P(\mathrm{PP}|\mathrm{AP})\times P(\mathrm{AP})}{P(\mathrm{PP})} \end{array}$$

where AP and PP represent the *actual positive* and the *predicted positive* events, respectively. Stated otherwise, the lower the probability of observing a positive instance, the lower the value of $P\left(\mathrm{AP}\right)$, which results in a lower Precision value $P\left(\mathrm{AP}|\mathrm{PP}\right)$. Using historical data, we then calculate the expected month-by-month probability of observing a rain event. By plugging these probabilities into (13), we can obtain the results shown in the bottom plot of Figure 6, which displays the monthly probabilities along with the expected Precision of the ensemble model over the course of the year. The probability of rainfall ranges from as low as 0.02% in July to nearly 9% in December.

### 4.2. Regression

Starting from the observations and results obtained in the analysis and building of the classification model, we proceeded with the regression task by training our model directly on a rain-conditioned dataset, as this yielded superior performance. We adopted the same systematic experimentation and evaluation approach used for classification, using as target variable the RR measured value. This variable shows a long right tail and approximately follows a log-normal distribution, which, combined with a large number of zero values, makes the regression non-trivial.

#### 4.2.1. Conditional Piecewise Ensemble Regressor

Following the same procedure as for the classification task, we conducted experiments with several models and neural architectures. Unlike the classification task, the neural networks did not bring any advantages compared to traditional machine learning models. We report the validation performance of various models in Table 8, where we can see that a linear regression can already solve the regression task with good performance. This is in line with the literature, where the rainfall estimation is performed by leveraging the linear relationship between rainfall and attenuation, as mentioned in Section 2. On the validation set, the best-performing model was the GBM trained on all the aggregated statistics, while other models such as the GRU trained on the past 60 min raw data and the MH-NN did not show any improvement. We also found that ensemble models built on them did not bring superior performance.

Moved by these considerations, we eventually used the GBM model, which consistently emerged as the optimal model, and built a conditional ensemble, an architecture in which a regressor and a classifier are jointly used [46]. In more detail, the predicted continuous output of the regressor is transformed through a parametrized piecewise function, leveraging the output probability of the classifier and mapping out the predictions falling below a fixed value. This function can be written as

(14) $$\begin{array}{c} f_{i}\left(x\right)=\left\{\begin{array}{cc} 0, & \mathrm{if}\;y_{\mathrm{class},i}<x\\ y_{\mathrm{regr},i}, & \mathrm{otherwise} \end{array}\right.\;, \end{array}$$

where, for every temporal instance *i*, $y_{\mathrm{regr},i}$ and $y_{\mathrm{class},i}$ are, respectively, the regressor and classifier output of the *i*th observation, while *x* is the conditional threshold which is a tunable hyperparameter optimized during the model-training phase (with the classifier’s 65% decision threshold found in Section 4.1.3).

**Figure 7:**
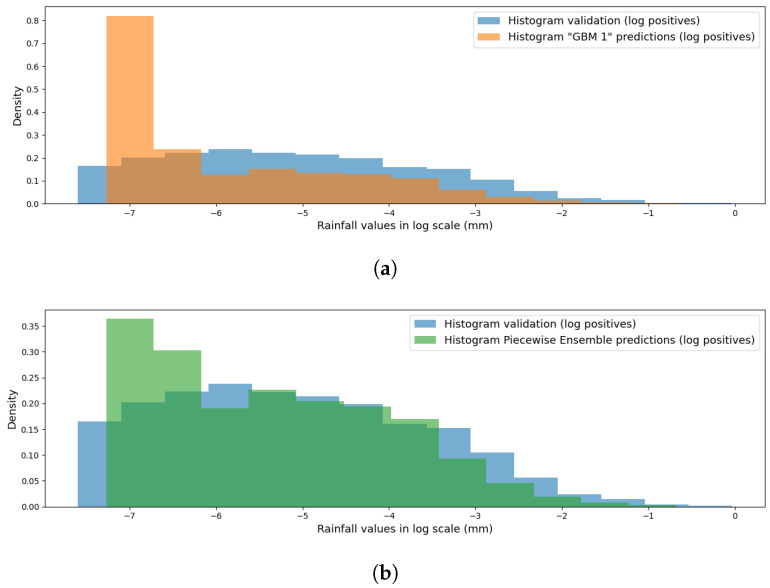
(**a**) Histogram of the positive prediction of the GBM model (orange) compared to the actual distribution (cyan). (**b**) Same plot, but comparing the conditional piecewise ensemble model (green).

The two models used for the conditional ensemble are the best-performing regressor and classifier on the validation set observed so far, namely, the GBM regressor and the ensemble classifier composed of a GRU and an MH-NN previously built in Section 4.1. The results obtained on the validation set are reported in the last row of Table 8, where it can be noticed that the performance showed a modest but consistent improvement with respect to those reached by the GBM model alone. The reason lies in the better prediction power at small rainfall values, as can be noticed by looking at Figure 7: the GBM regressor has a high error margin on small values, as it fails to predict zero and instead predicts extremely small values. This is due to its loss function, which is not sensitive to such small errors. On the other hand, the conditional ensemble reduced the overall error by better approximating the target distribution, forcing these extreme small values to zero thanks to the information given by the classifier.

#### 4.2.2. Model Assessment on Test Data

**Figure 8:**
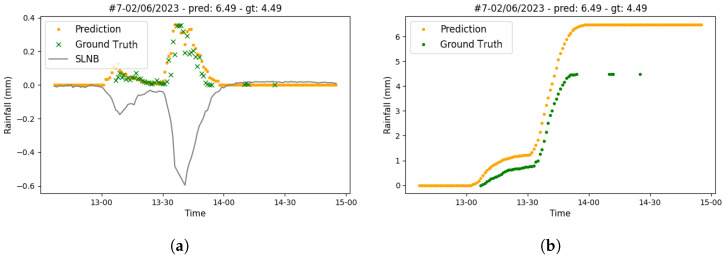
Example of model predictions on a single rain event on the test dataset against actual values (green crosses) for the location of Rome. (**a**) Minute-by minute prediction (orange dots) against actual values (green crosses) and raw SNLB signal (black line) (**b**) Cumulative prediction (orange dots) against actual values (green dots).

After using the validation data set for model selection, we used the test set for model assessment. In Table 9, we report the performance of the conditional piecewise ensemble model on the test set. Overall, the test results are in line with the validation, albeit the validation errors are slightly lower than the test ones. The NMAE in all instances amounts to 0.75, which is slightly higher than the validation one at 0.65 (see Table 8). Similarly, the NMAE on rain instances progressed from 1.4 to 1.5 for the validation and test sets, respectively. We believe this slightly higher error rate in the test compared to the validation is a reflection of a subtle shift in the distribution of rainfall between the validation and test set, a plausible scenario considering that the validation set was mainly winter and spring months whilst the test set contained the summer months. A visualization of the predictions obtained on a single rain event is given in Figure 8.

**Figure 9:**
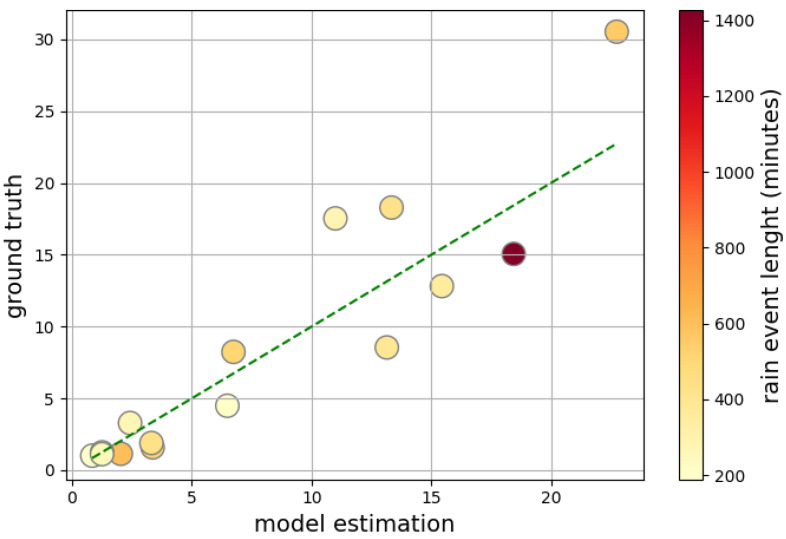
Scatter plot of the cumulative rainfall predictions on test rain events against the event actual value, for the location of Rome and using the conditional piecewise ensemble model. The dashed line is the bisector that represents the perfect model estimation relative to the ground truth.

Finally, we provided a generalization analysis by computing the performance metrics on the cumulative rainfall of the single rain events across different locations. We applied our conditional ensemble model to the test data of all the available locations, obtaining the results reported in Table 10. The MAE for all events is equal to 2.6, 2.2, and 3.7 millimeters of rain for Rome, Pisa, and Massa, respectively. The NMAE metric multiplied by 100, which provides the average error as a percentage of the range of the target value, is essentially stable across all the locations at approximately 11%. Other metrics mirror the same trend as MAE and also show similar performance throughout the locations. In particular, the correlation metric, which measures the extent to which the value predicted by the model fits with the actual value, is essentially constant. In Figure 9, we show the scatter plot obtained by plotting the predictions and the true measures for the cumulative precipitation of all test events in the Rome location. As anticipated by the high correlation value obtained in Table 10, we can see a clear linear dependence between the predicted and actual rainfall amount of the events. Finally, the results obtained from our model demonstrate a significant improvement over a state-of-the-art power-law-based algorithm, as implemented on the same data type and defined events, as reported in [37]. The authors reported an RMSE of 7.58 across all sites, while our model achieved an RMSE of 4.112 (Table 10), representing a 45.8% reduction in error compared to the power-law-based algorithm.

## 5. Discussion and Conclusions

We investigated ML capabilities for end-to-end rainfall estimation derived from satellite downlink attenuation, a domain in which, to our knowledge, there has not been a significant end-to-end ML implementation so far. The research was split into two main research topics associated with our task: rainfall detection and rainfall estimation, which, in ML terms, were addressed by building, respectively, a classifier and a regressor. We took advantage of the possibilities given by the NEFOCAST platform, which provided data from several locations (Rome, Pisa, and Massa) with both measures of SNR and actual rain measures.

For the classification segment, we found that complex neural network structures, like GRU and MH-NN, outperform simpler ML models such as GBM. We also utilized ensemble methods, which significantly enhanced performance. Regarding regression performance, we achieved robust results and found that advanced neural network architectures did not enhance performance compared to tree-based ML models using extracted statistical features. This pattern essentially tells us that, while complex dynamics in the signal can be exploited for classification, once rainfall is confirmed, the signal-to-rainfall relationship adheres to the less complex dominant contributions described by physical models documented in the literature. Hence, straightforward models like GBM perform relatively well, while advanced models do not offer significant advantages. Finally, we experimented with incorporating the classifier probability output into the regression task, eventually defining a conditional piecewise ensemble model, a targeted method to leverage the classifier output, which did boost performance, especially in reducing errors from the regressor for dry instances. Overall, we obtained a well-performing minute-by-minute regressor with an NMAE of 0.4 and 1.5 on dry and rainy instances, respectively.

Model assessment results show that the performance obtained when testing the model trained on data from Rome, where we took advantage of the accurate disdrometer measures, is balanced across different locations. These findings suggest that the implemented preprocessing steps are generic and that the model has successfully identified relevant patterns related to rain dynamics. Overall, our model generalizes well to new data from the other two SLNB locations (Pisa and Massa), achieving an NMAE of approximately 11% for cumulative rain event predictions across all locations. Finally, the merit parameters obtained in this paper demonstrate a better agreement of the ML model rainfall estimates with the measured precipitation compared to those obtained with the power-law-based method.

We have identified two possible areas for improvement in future work. First, it would be beneficial to further enhance the preprocessing techniques by reducing noise and improving signal quality during heavy rain events. These steps could drastically improve the results by providing a cleaner input signal-to-noise ratio for the models, allowing them to better capture complex or hidden patterns in high-precipitation instances. In addition, we plan to generalize the training using data from different locations by acquiring the time series of co-located disdrometer and SLNB data, exploring the advantages of integrating datasets from various locations. Ultimately, a thorough generalization analysis should be conducted by analyzing the inference performance on different geographical locations along with different input signal rates (Ku-band, K-band, etc.) to evaluate the potential for the proposed method to be applied in a wider range of contexts.

## Figures and Tables

**Table 1:** NEFOCAST locations with available devices and related temporal availability of data.

Location	Device	Available Timeframe
Rome	SLNB	16/07/2021–11/05/2023
	TBRG	21/03/2022–25/05/2023
	Disdrometer	30/06/2021–18/05/2023
Pisa	SLNB	08/07/2022–12/05/2023
	TBRG	08/07/2022–12/05/2023
Massa	SLNB	17/07/2021–11/05/2023
	TBRG	30/06/2021–18/05/2023

**Table 2:** GRU model architecture and grid-search parameters. dp_rate refers to the drop-out rate, which was bounded between 0 and 60%.

	Data Input Shape	Hyperparameters			
		**# Layers**	**# Units/Layer**	**dp_Rate/Layer**	**# Parameters**
Grid-Search range	(43k, 60)	GRU: (1, 9, step = 1) Dense: (0,4, step = 1)	GRU: (32, 128, step = 32) Dense: (16, 128, step = 32)	[0, 0.6]	-
Tuned GRU classifier	(43k, 60)	6 GRU 1 Dense	GRU: (96 × 6), Dense: (16)	(0.33)	270k

**Table 3:** MultiHeadNN model architecture and grid-search parameters.

	Data Input Shape	Hyperparameters			
		**# Layers**	**# Units/Layer**	**dp_Rate/Layer**	**# Parameters**
Grid-Search range	(57k, 60, 37)	Head: GRU: (1, 5, step = 1) Dense: (1, 5, step = 1) Out: Dense: (0, 6, step = 1)	Head: GRU: (32, 128, step = 32) Dense: (64, 256, step = 32) Out: Dense: (16, 256, step = 32)	[0, 0.6]	-
Tuned MultiHeadNN classifier	(57k, 60, 37)	Head: (8 GRU, 6 Dense) Out: 1 Dense	Head: ((96 × 2, 32 × 2, 128, 64 × 3) (192, 128, 128, 192, 160, 64)) Out: (128)	(0.28)	386k

**Table 4:** Validation metrics of the best-performing models on a rain-conditioned dataset, with a training–validation imbalance of 50% and with decision threshold optimized for the highest F1-score.

Model	Validation Metrics				
	AUC–ROC	Accuracy	F1-Score	Precision	Recall
GBM	0.909	0.833	0.804	0.796	0.812
GRU	0.915	0.831	0.808	0.778	0.842
MultiHeadNN	0.916	0.832	0.802	0.781	0.807
Ensemble	0.918	0.836	0.818	0.771	0.871

**Table 5:** Validation metrics of the best performing models on the original dataset with decision threshold optimized for the highest F1-score.

Model	Validation Metrics				
	AUC–ROC	Accuracy	F1-Score	Precision	Recall
GBM	0.946	0.974	0.699	0.703	0.696
GRU	0.968	0.977	0.715	0.762	0.674
MultiHeadNN	0.968	0.976	0.720	0.734	0.706
Ensemble	0.969	0.977	0.727	0.745	0.710

**Table 6:** Comparison of ensemble model performance with different threshold levels.

Metric	Decision Threshold		
	0.65	0.54	0.42
Recall	0.750	0.800	0.850
FPR	0.016	0.026	0.043
Precision	0.672	0.585	0.470
F1-Score	0.710	0.675	0.605

**Table 7:** Test metrics of the ensemble model with a decision threshold of 65%.

Metric	Value
AUC–ROC	0.975
Accuracy	0.979
Precision	0.613
Recall	0.809
F1-Score	0.698
FPR	0.043

**Table 8:** Validation performance of the best-performing regression models on the original unbalanced dataset.

Model	Metrics	
	NMAE (Rain Instances)	NMAE (All Instances)
Linear Regression	1.512	0.772
Random Forest	1.484	0.711
GBM	1.411	0.698
Conditional Ensemble	1.406	0.647

**Table 9:** Test metrics of the conditional piecewise ensemble model. NMAE on dry instance is the error when the true value is zero (no rain instances).

Metric	Instances	Value
NMAE	all	0.748
NMAE	dry	0.359
NMAE	rain	1.537

**Table 10:** Assessment metric of cumulative rainfall prediction on test rain events for all locations using the conditional piecewise ensemble model.

Location	Test Metrics on Cumulative Data			
	Correlation	RMSE	MAE	NMAE × 100 (%)
Rome	0.920	3.468	2.576	11.763
Pisa	0.923	4.172	2.209	10.460
Massa	0.931	4.696	3.712	11.420
All sites	0.924	4.112	2.832	11.214

## Data Availability

The original contributions presented in the study are included in the article; further inquiries can be directed to the corresponding author.

## Abbreviations

The following abbreviations are used in this manuscript:

AUC	area under ROC curve
CENN	conditional ensemble neural network
CML	commercial microwave link
DL	deep learning
DNN	dense neural network
ERM	empirical risk minimization
ESNDR	energy-per-symbol-to-noise density ratio
FF-DNN	feed-forward dense neural network
FPR	false positive rate
GBM	gradient boosting machine
GRU	gated recurrent unit
GS	ground station
GT	ground truth
IPL	ice particles layer
IoT	internet of things
KNN	k-nearest neighbors
LL	liquid layer
LSTM	long short-term memory
MAE	mean absolute error
MAPE	mean absolute percentage error
MHNN	multi-head neural network
ML	machine learning
NB	normalized bias
NMAE	normalized mean absolute error
NMB	normalized mean bias
PL	power-law
RF	radio frequency
RMSE	root mean squared error
RNN	recurrent neural network
ROC	receiver operating characteristic
RR	rain rate
SLNB	SmartLNB
SML	satellite-to-earth microwave link
SNR	signal-to-noise ratio
SVM	support vector machine
TBRG	tipping bucket rain gauge
TCN	temporal convolutional network
TPR	true positive rate
UT	user terminal
